# The Anti-Cancer Potential of Genistein: Single-Cell RNA Sequencing Analysis and Spatial Transcriptome Reveal That Genistein Targets HSD17B1 to Inhibit the Progression of Gastric Adenocarcinoma

**DOI:** 10.3390/ijms262110369

**Published:** 2025-10-24

**Authors:** Xianbing Wang, Junyuan Zhang, Jiaying Jiang, Yi Wang

**Affiliations:** 1 The School of Molecular Sciences, The University of Western Australia, Perth, WA 6009, Australia; 24474327@student.uwa.edu.au (X.W.); 24473518@student.uwa.edu.au (J.Z.); 2 Biological Science Research Center, Southwest University, Chongqing 400715, China; 3 The State Key Laboratory of Resource Insects, Southwest University, Chongqing 400715, China; swuer1@email.swu.edu.cn

**Keywords:** gastric adenocarcinoma, genistein, single-cell RNA sequencing analysis, molecular docking, spatial transcriptome

## Abstract

Genistein has anti-cancer effects, but its molecular targets in gastric adenocarcinoma (GA) are unclear. This study used single-cell RNA sequencing (scRNA-seq) and spatial transcriptomics (ST) to explore genistein’s “drug-gene-cell” interactions in GA. GA- and genistein-related target genes were retrieved and intersected with differentially expressed genes identified from bulk transcriptomic data. Machine learning screened candidates, and survival analysis assessed prognosis. Molecular docking with genistein validated key genes, with molecular dynamics assessing binding stability. *HSD17B1*, *EZH2*, *CCNB1*, *CCNB2*, *CDKN2A*, and *IGFBP6* were identified as key candidate genes with prognostic value for GA. Specifically, samples in the *IGFBP6* high-expression group were associated with higher survival probability, whereas the opposite trend was observed for the other five genes. In addition, HSD17B1 was genistein’s main target in GA treatment, showing a strong binding affinity with genistein (binding energy of −8.1 kcal/mol). scRNA-seq analysis indicated that *HSD17B1* was predominantly expressed in epithelial cells and was significantly involved during their malignant transformation (confirmed by ST). This study identified *HSD17B1* as a critical target gene for genistein in GA treatment, emphasizing its roles in the malignant transformation of epithelial cells, thus providing a theoretical foundation for understanding the therapeutic mechanism of genistein in GA.

## 1. Introduction

Gastric cancer (GC) is the fifth most common and lethal gastrointestinal malignancy globally. According to the American Cancer Society, over 968,000 new GC cases were reported worldwide in 2022, resulting in nearly 660,000 deaths [1]. Gastric adenocarcinoma (GA) is the predominant subtype, accounting for 90% to 95% of GC cases. For patients with advanced-stage GA (clinical stage IV), the median overall survival is between 9 and 10 months, with a 5-year survival rate of less than 30% [2,3,4]. Despite its high incidence, research on therapeutic strategies for GA remains limited compared to other cancers.

Phytoestrogens, as naturally occurring bioactive compounds, have long been recognized for their antioxidant properties and potential role in cancer prevention [5]. These compounds share structural similarities with endogenous estrogen, enabling them to modulate estrogen receptor (ER) expression and potentially induce apoptosis through ER downregulation—a mechanism that has shown promise in breast cancer therapy [5]. Although the specific effects of phytoestrogens in GA have not yet been systematically investigated, emerging evidence from a cross-sectional study indicates a significant association between phytoestrogens and lung function, with this relationship appearing to be influenced by phytoestrogen subtypes, patient sex, and smoking status [6].

Genistein, or 5,7,4′-trihydroxyisoflavone, is a naturally occurring isoflavone (phytoestrogens) primarily found in the roots and seeds of leguminous plants. Genistein, though not a steroidal estrogen like estradiol, estriol, and estrone, shares structural similarities with them such as possessing aromatic rings and phenolic hydroxyl groups that enable interactions with estrogen receptors (Figure S1). This compound has demonstrated significant anti-inflammatory and anti-cancer effects across various malignancies, including colorectal, bladder, breast, prostate, and non-small cell lung cancers [7]. It inhibits the secretion of angiogenic factors under hypoxic conditions, thereby limiting tumor growth [8]. Additionally, genistein reduces cancer stem cell-like properties in GC cells, decreasing their self-renewal, drug resistance, and invasive capabilities [9,10]. Moreover, it enhances the therapeutic efficacy of conventional chemotherapeutic agents such as 5-fluorouracil (5-FU) and cisplatin, and sensitizes TRAIL-resistant GA cells to TRAIL-induced apoptosis [11,12]. In GA, genistein inhibits cellular proliferation by inducing cell cycle arrest, suppressing NF-κB activity, and upregulating apoptosis-associated proteins like caspase-3, thereby promoting dose- and time-dependent apoptosis in cancer cells [13,14,15]. However, the precise molecular target of genistein remains incompletely defined, and the underlying mechanisms of its pharmacological action require further exploration.

Single-cell RNA sequencing (scRNA-seq) is an advanced, high-throughput technique that enables transcriptomic profiling at the individual cell level, providing insights into gene expression heterogeneity within cell populations. This method offers significant advantages, including the ability to identify cellular diversity, reconstruct differentiation pathways, and detect rare cell types [16]. scRNA-seq is widely utilized in GA research to examine tumor cell diversity at the single-cell level, identifying various immune cell subsets, cancer-associated fibroblasts, and malignant epithelial groups. It also aids in reconstructing cell differentiation trajectories and uncovering key transcription factors and signaling pathways involved in tumor progression. These insights are instrumental in pinpointing potential immunotherapy targets and developing personalized treatment strategies [17,18]. Additionally, spatial transcriptomics (ST), which combines scRNA-seq with spatial localization, allows for the concurrent collection of transcriptomic and positional information from tissue sections. This approach enhances the study of the tissue microenvironment, cellular diversity, and spatial regulatory mechanisms with greater resolution and context [19,20]. Chen MM et al. combined ST with single-nucleus RNA sequencing to analyze 25 pancreatic ductal adenocarcinoma samples, clarifying the spatial niche differences in tumor microenvironment, neuro-related cell composition and interactions between high/low neural invasion cases, and providing spatial evidence for deciphering tumor-immune-nerve crosstalk and neural invasion mechanisms [21]. Similarly, the Ma H team integrated ST and scRNA-seq data from multiple GA samples and identified intratumoral heterogeneity based on spatially resolved gene expression patterns, which were associated with distinct immune microenvironments [22]. In this study, scRNA-seq and ST were employed to explore cellular heterogeneity in GA, with the goal of identifying key functional cell populations and disease-related genes, along with their spatial expression patterns within the tumor microenvironment.

**Figure 1:**
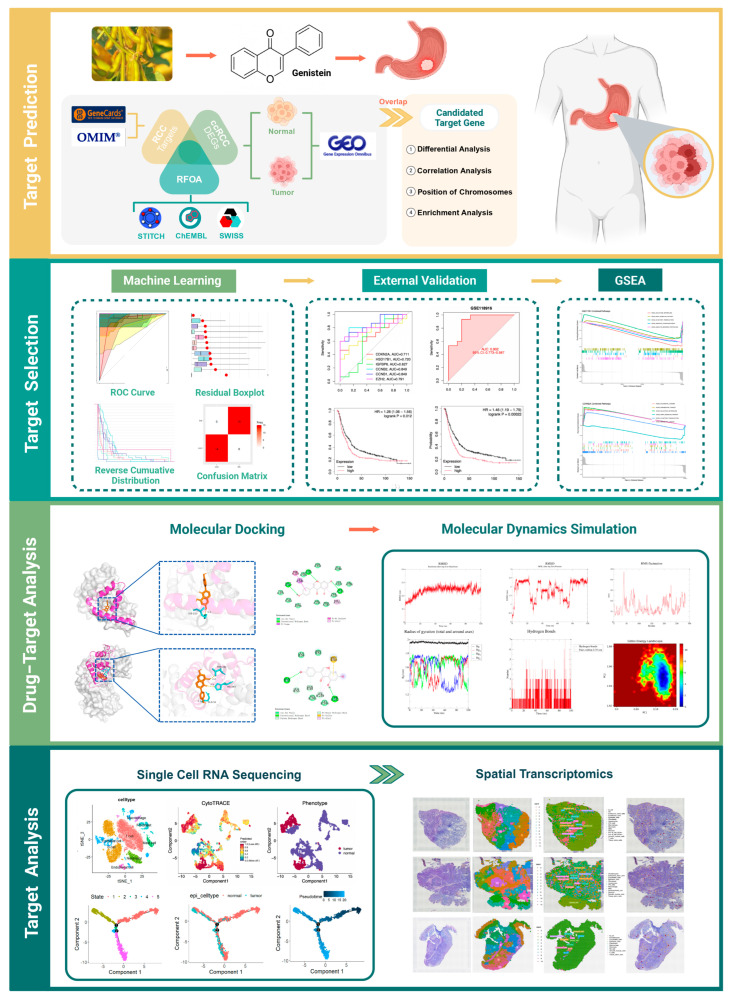
Study design.

This study integrated network pharmacology, bulk RNA-seq, and molecular docking to identify key genes targeted by genistein in GA, supported by molecular dynamics (MD) simulations. scRNA-seq and ST further characterized tumor heterogeneity and the microenvironment, revealing critical cell populations and spatially resolved gene expression patterns (Figure 1).

## 2. Results

### 2.1. The Candidate Genes Were Involved in Immune and Cancer-Related Pathways

**Figure 2:**
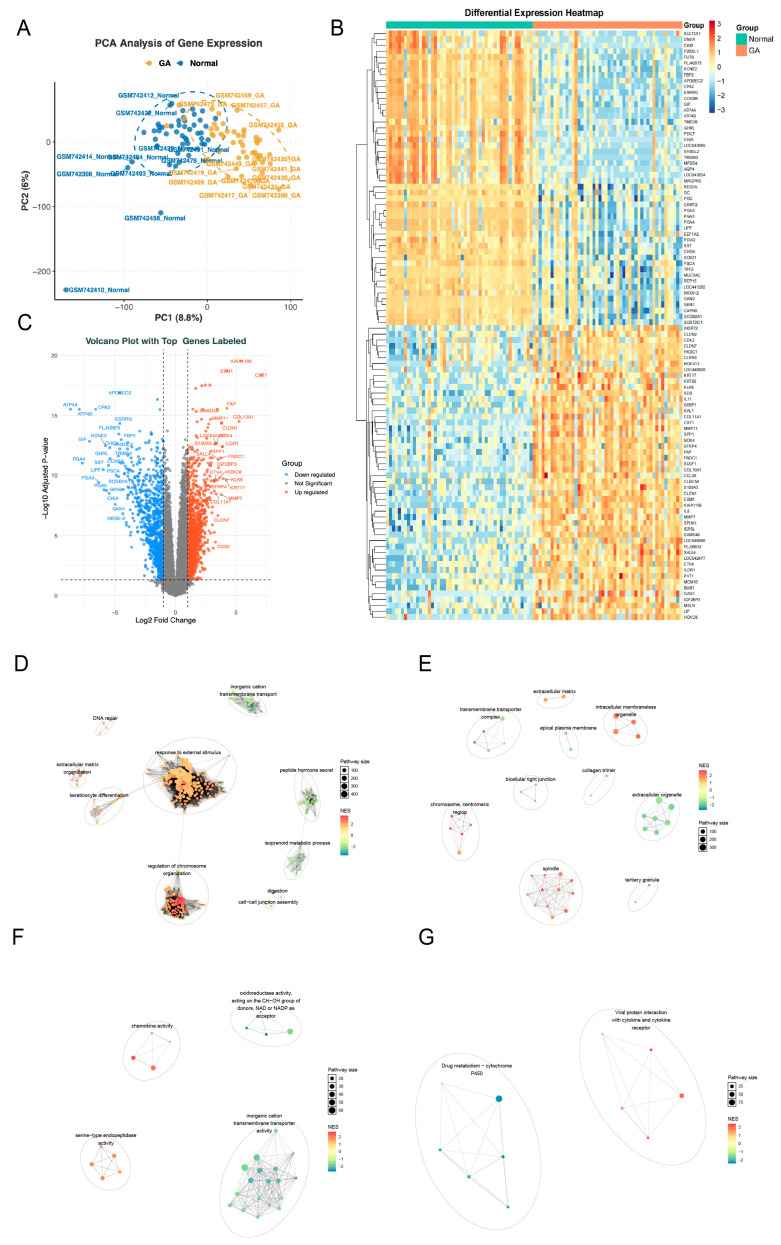
Differential expression and functional enrichment analyses in the GSE29998 dataset. (**A**) Principal component analysis (PCA) results of samples in the GSE29998 dataset. (**B**) Heatmap of differentially expressed genes (DEGs) between GA and the control groups fom the GSE29998 dataset. (**C**) Volcano plot of DEGs between the GA group and the control group in the GSE29998 dataset. (**D**–**G**) Cluster annotation results of Gene Ontology (GO) terms and Kyoto Encyclopedia of Genes and Genomes (KEGG) pathways enriched by DEGs. (**D**), Biological Process (BP); (**E**), Cellular Component; (**F**), Molecular Function; (**G**), KEGG.

Principal components analysis (PCA) of the GSE29998 dataset revealed a distinct separation between GA and control samples (Figure 2A). Differential expression analysis identified 2043 differentially expressed genes (DEGs), including 904 downregulated genes and 1139 upregulated genes in GA samples (Figure 2B,C). Pathway enrichment analysis showed that Gene Ontology (GO) terms were mainly enriched in stress response, chromosome organization, extracellular structure, immune activity, and ion transport, while Kyoto Encyclopedia of Genes and Genomes (KEGG) pathways highlighted drug metabolism (cytochrome P450) and viral protein–cytokine interactions, indicating coordinated regulation of cellular response, immunity, and metabolism (Figure 2D–G).

**Figure 3:**
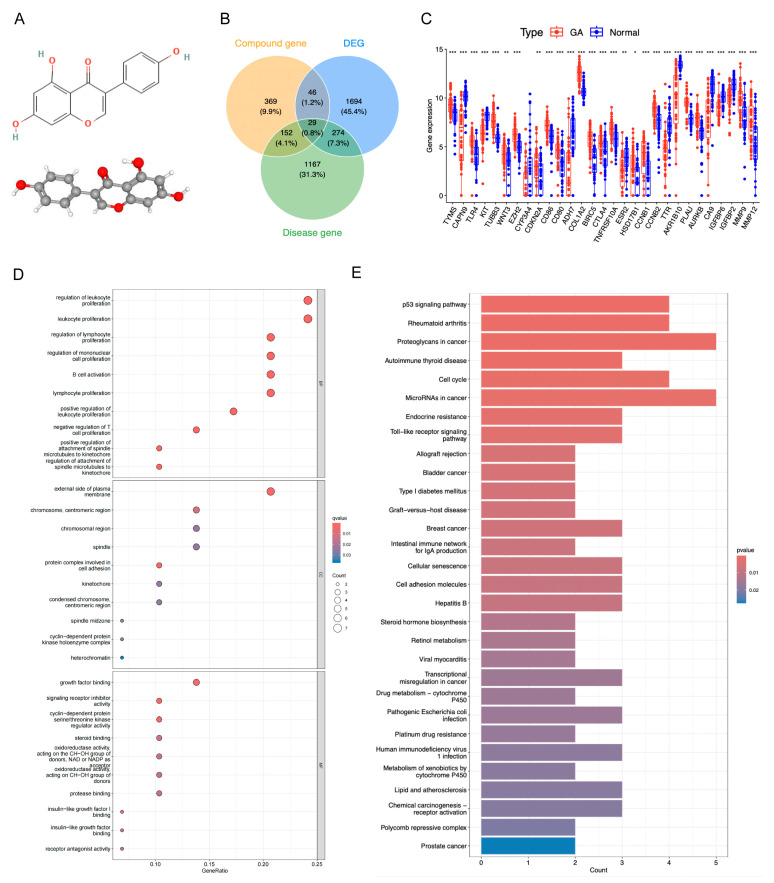
Acquisition and functional analysis of candidate genes. (**A**) The molecular structure of genistein. (**B**) Venn diagram of drug target genes, GA target genes, and DEGs to identify candidate genes. (**C**) Expression box plot of candidate genes in GSE29998 dataset. * *p* < 0.05, ** *p* < 0.01, *** *p* < 0.001. (**D**) GO terms enriched by candidate genes. (**E**) KEGG pathways enriched by candidate genes.

The molecular structure of genistein is shown in Figure 3A. After deduplication and merging, genistein was predicted to interact with 596 drug target genes, while GA was associated with 1622 target genes. The intersection of drug target genes, GA target genes, and DEGs resulted in 29 candidate genes, including 18 upregulated genes (e.g., *TYMS* and *TLR4*) and 11 downregulated genes (e.g., *KIT* and *CAPN9*) (Figure 3B,C, Table S1). Chromosomal localization analysis revealed that *EZH2*, *AKR1B10*, *COL1A2*, and *CYP3A4* were co-localized on chromosome 7 (Figure S2A). Correlation analysis indicated that most genes exhibited positive correlations, exemplified by *TYMS* and *CCNB2* (Figure S2B). The protein–protein interaction (PPI) network demonstrated complex interactions among these genes at the protein level, such as *CA9* interacting with *CD28* (Figure S2C). GO annotation revealed significant enrichment of these candidate genes in 451 biological process (BP) terms, 22 molecular function (MF) terms, and 12 cellular component (CC) terms, with notable immune-related functions such as leukocyte proliferation, B cell activation, and lymphocyte proliferation (Figure 3D). KEGG pathway enrichment identified 38 significantly enriched pathways, including cancer-related pathways such as the p53 signaling pathway and proteoglycans in cancer (Figure 3E).

### 2.2. HSD17B1, EZH2, CCNB1, CCNB2, CDKN2A, and IGFBP6 Served as Candidate Key Genes

**Figure 4:**
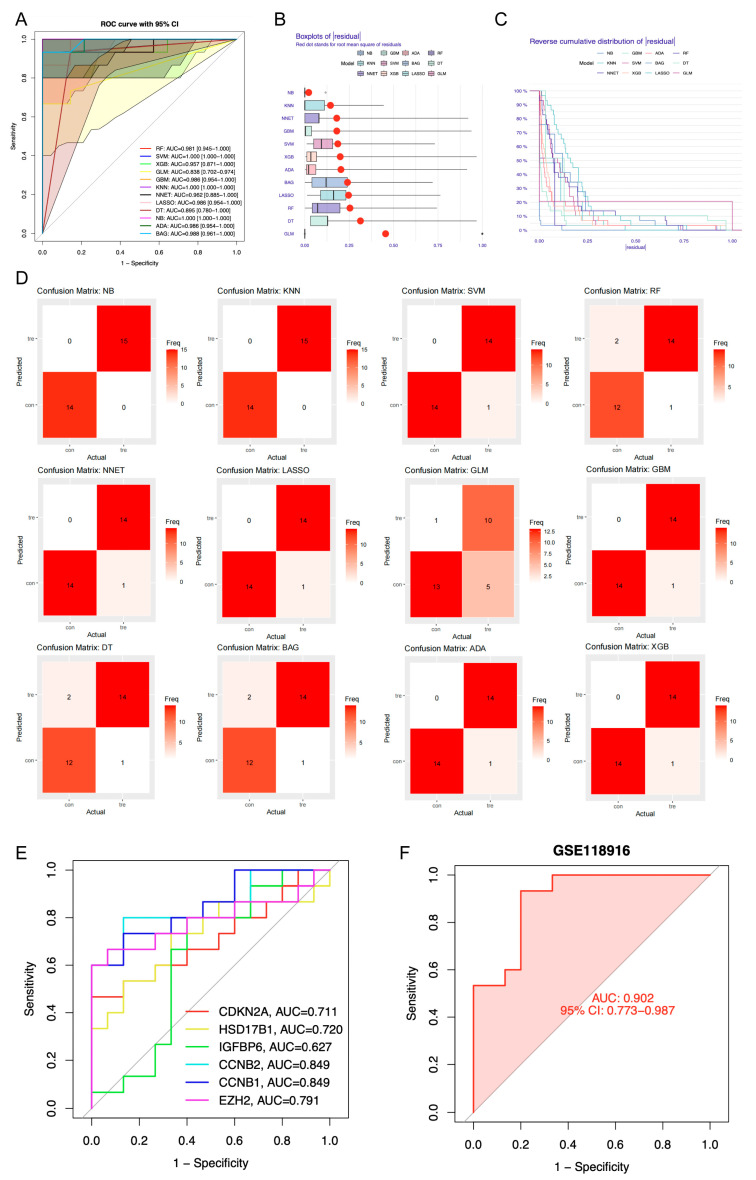
Identification of candidate key genes by machine learning algorithms. (**A**) Receiver Operating Characteristic (ROC) curves of 12 distinct machine learning algorithms. RF, Random Forest; SVM, Support Vector Machine; XGB, Extreme Gradient Boosting; GLM, Generalized Linear Model; GBM, Gradient Boosting Machine; KNN, k-Nearest Neighbors; NNET, Neural Network; LASSO, Least Absolute Shrinkage and Selection Operator; DT, Decision Tree; NB, Naive Bayes; ADA, Adaptive Boosting; BAG, Bootstrap Aggregating. (**B**) Residual box plot of 12 distinct machine learning algorithms. (**C**) Reverse cumulative distribution graph of 12 distinct machine learning algorithms. (**D**) Confusion matrix of 12 distinct machine learning algorithms. (**E**) ROC curves of 6 candidate key genes. AUC, area under the curve. (**F**) ROC curves of predictive model constructed from 6 candidate key genes within the GSE118916 dataset.

Among the machine learning models developed using candidate genes, the NB model showed superior performance, with the highest area under the curve (AUC) value (AUC = 1), minimal residual error, and greatest classification accuracy as indicated by the confusion matrix (Figure 4A–D, Table S2). Therefore, the Naive Bayes (NB) model was selected as the optimal predictive model, with 6 genes within the model identified as candidate key genes (*HSD17B1*, *EZH2*, *CCNB1*, *CCNB2*, *CDKN2A*, and *IGFBP6*). These candidate genes demonstrated high diagnostic accuracy for GA, with AUC values above 0.6 for each individual gene. A predictive model constructed from these genes in the GSE118916 dataset yielded an AUC greater than 0.9 (Figure 4E,F). Kaplan–Meier survival analysis revealed significant differences in survival outcomes among the expression groups of all candidate key genes (Figure S3).

### 2.3. Functional Enrichment Analysis of the Candidate Key Genes

**Figure 5:**
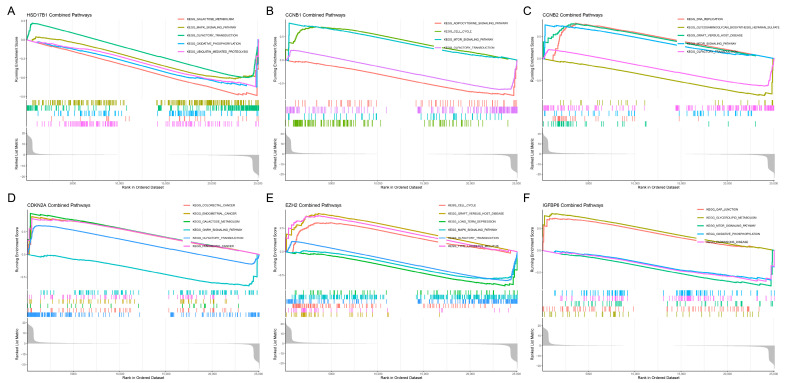
Gene set enrichment analysis (GSEA) of candidate key genes. (**A**) *HSD17B1*, (**B**) *CCNB1*, (**C**) *CCNB2*, (**D**) *CDKN2A*, (**E**) *EZH2*, (**F**) *IGFBP6*.

To further explore the biological functions and potential mechanisms of the six hub genes, Gene Set Enrichment Analysis (GSEA) was performed using KEGG pathway data (Figure 5A–F). The analysis revealed significant enrichment of cell cycle-related pathways in multiple genes, including *CCNB1*, *CCNB2*, and *EZH2*, suggesting their involvement in regulating cell proliferation. *CDKN2A* was associated with several cancer-related pathways, including colorectal cancer, endometrial cancer, and pancreatic cancer, while *HSD17B1* was enriched in metabolic and signaling pathways, such as galactose metabolism and MAPK signaling. *IGFBP6* was primarily linked to gap junctions, glycerolipid metabolism, and mTOR signaling pathways. Notably, the cell cycle, olfactory transduction, and MAPK signaling pathways were recurrently enriched across several genes, indicating their potential role in tumor progression.

### 2.4. HSD17B1 Was Treated as a Key Gene

**Figure 6:**
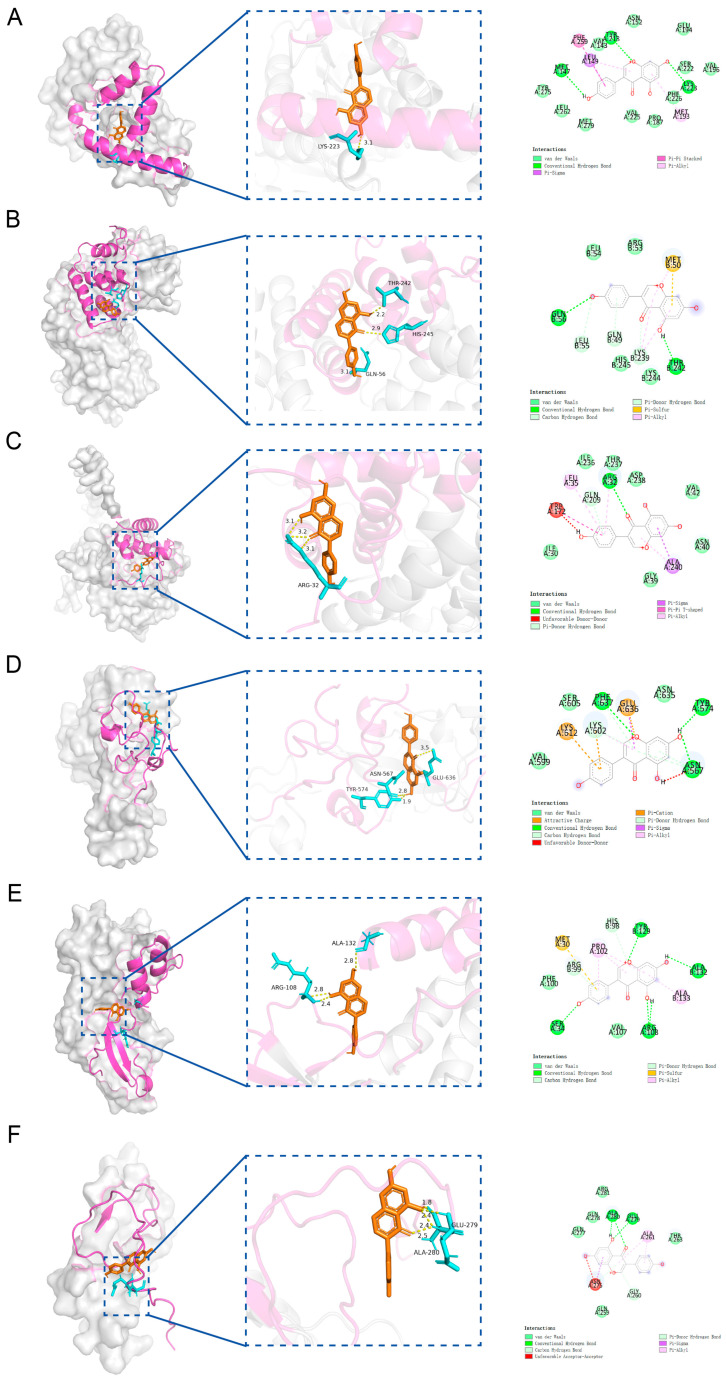
Molecular docking of 6 candidate key genes and genistein. (**A**) Molecular docking between HSD17B1 and genistein. (**B**) Molecular docking between CCNB1 and genistein. (**C**) Molecular docking between CCNB2 and genistein. (**D**) Molecular docking between EZH2 and genistein. (**E**) Molecular docking between CDKN2A and genistein. (**F**) Molecular docking between IGFBP6 and genistein.

**Figure 7:**
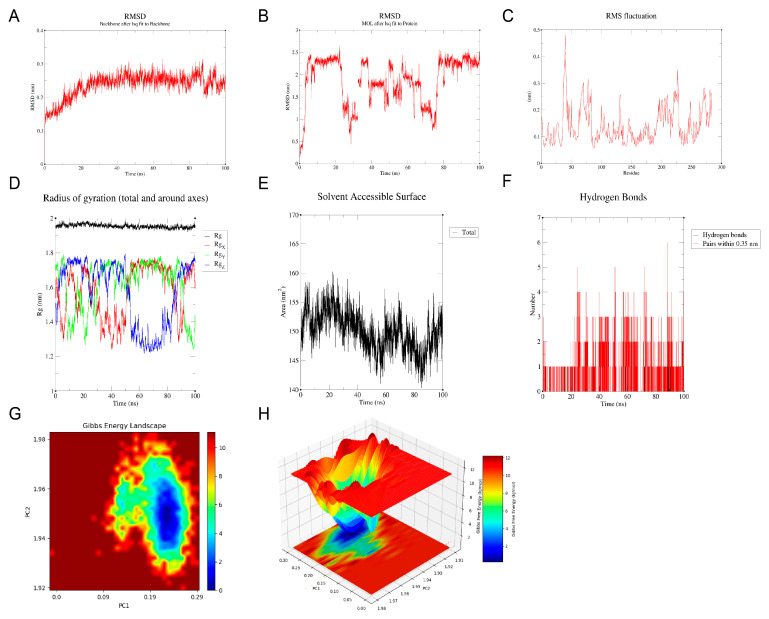
Molecular dynamics (MD) simulation of HSD17B1 and genistein complex. (**A**,**B**) root mean square deviation (RMSD) of HSD17B1 and genistein complex. (**C**) Root mean square fluctuation (RMSF) of HSD17B1 and genistein complex. (**D**) Radius of gyration (Rg) of HSD17B1 and genistein complex. (**E**) Buried solvent accessible surface area (buried SASA) of HSD17B1 and genistein complex. (**F**) Hydrogen bond counts of HSD17B1 and genistein complex. (**G**,**H**) Gibbs energy landscape of HSD17B1 and genistein complex.

Molecular docking analysis between the candidate genes and genistein showed binding energies consistently below −5 kcal/mol, indicating a strong potential for molecular interaction (Figure 6A–F, Table S3). Specifically, genistein formed multiple hydrogen bonds with the amino acid residues of the proteins encoded by the candidate genes, with corresponding binding energies as follows: HSD17B1 (−8.1 kcal/mol), EZH2 (−7.3 kcal/mol), CCNB1 (−6.6 kcal/mol), CCNB2 (−8.0 kcal/mol), CDKN2A (−6.6 kcal/mol), and IGFBP6 (−6.7 kcal/mol). Since HSD17B1 exhibited the lowest docking energy with genistein, it was prioritized for MD simulation. The RMSD values of the HSD17B1-genistein complex were calculated to assess the stability of the complex structure during MD simulation (Figure 7A,B). The results indicated that after 20 ns of simulation, the RMSD values of the complex gradually stabilized, with fluctuations remaining below 0.1 nm, suggesting stable binding between HSD17B1 and genistein. Throughout the simulation, the RMS fluctuation, Rg, and buried SASA values of the HSD17B1-genistein complex converged, indicating structural stabilization. During the middle phase of the simulation, the complex displayed minimal RMSF variations, indicating improved residue-level stability (Figure 7C–E). The complex formed varying numbers of hydrogen bonds, ranging from 0 to 7, and the 2D and 3D Gibbs free energy landscapes revealed a distinct energy minimum along PC1 and PC2, collectively indicating a strong binding affinity (Figure 7F–H).

### 2.5. HSD17B1 Was Specifically Expressed in Epithelial Cells

**Figure 8:**
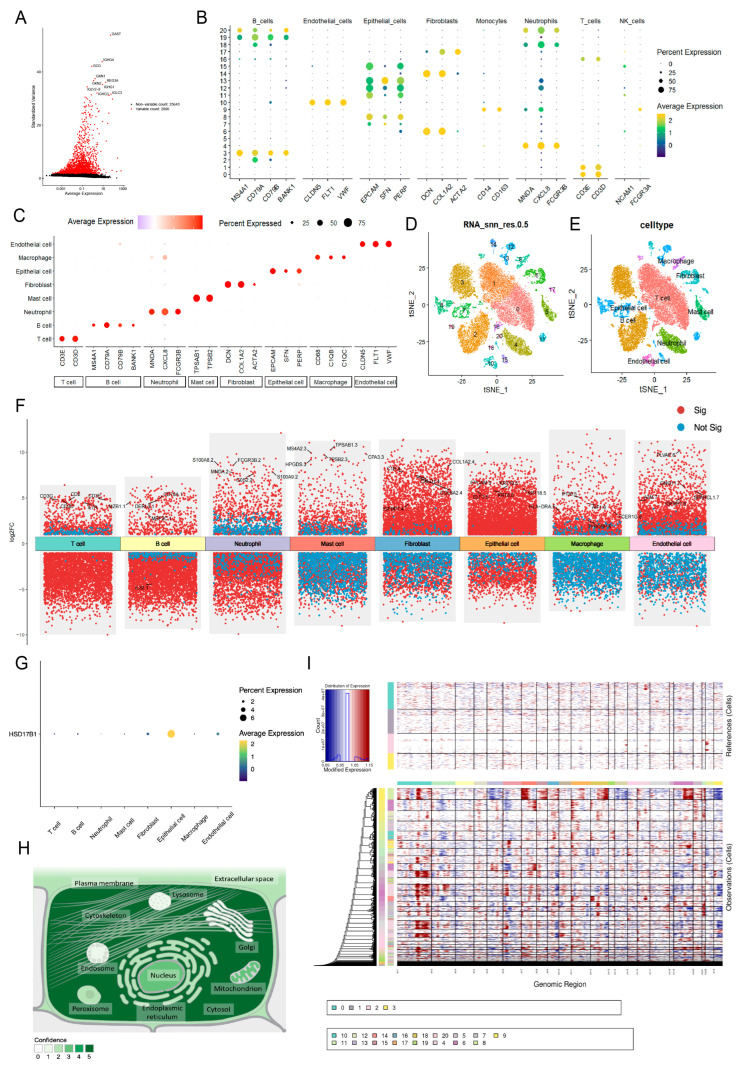
Identification of epithelial cells as key cell type. (**A**) Selection of top 2000 highly variable genes. (**B**–**C**) Expression of marker genes in cell types (**D**) UMAP clustering plot of 21 clusters in the GSE264203 dataset. (**E**) UMAP plot illustrating the distribution of 8 cell types. (**F**) Distribution of intergroup DEGs across cell types. (**G**) The expression of HSD17B1 in each type of cell. (**H**) Subcellular localization results of expression of HSD17B1. (**I**) inferCNV score evaluating pronounced copy number alterations (CNAs) for each cell cluster.

Following data filtration, a total of 28,682 cells and 27,643 genes from the GSE264203 dataset were retained for subsequent scRNA-seq analysis (Figure S4A,B). The top 2000 highly variable genes (HVGs) were selected for PCA, and the first 20 principal components (PCs) were used for further clustering analysis (Figure 8A). Thus, 21 clusters were identified and annotated into 8 cell types: mast cells, T cells, neutrophils, macrophages, fibroblasts, epithelial cells, endothelial cells, and B cells (Figure 8B–E and Figure S4C). Figure 8F illustrates the distribution of intergroup DEGs across cell types, with *HSD17B1* showing predominant expression in epithelial cells (Figure 8G). Subcellular localization analysis revealed that *HSD17B1* was primarily expressed in the cytosol (Figure 8H and Figure S4D). Integration of inferCNV analysis showed that epithelial cells (clusters 7, 13, and 15) exhibited pronounced copy number alterations (CNAs), suggesting potential epithelial–mesenchymal transition (EMT) within the tumor microenvironment (Figure 8I).

Enrichment analysis revealed mostly inverse correlations between the HALLMARK pathways and all cell types (Figure S5). Notably, a divergent positive correlation was observed between KRAS signaling-DN and cell types, with a particularly strong association in fibroblasts (R = 0.77), indicating aberrant activation of pro-proliferative intracellular signaling, potentially linked to oncogene activation. Conversely, the p53 signaling pathway was consistently downregulated across all cell populations, suggesting possible inactivation of tumor suppressor mechanisms.

### 2.6. HSD17B1 Was Mainly Expressed During the Malignant Transformation of Epithelial Cells

**Figure 9:**
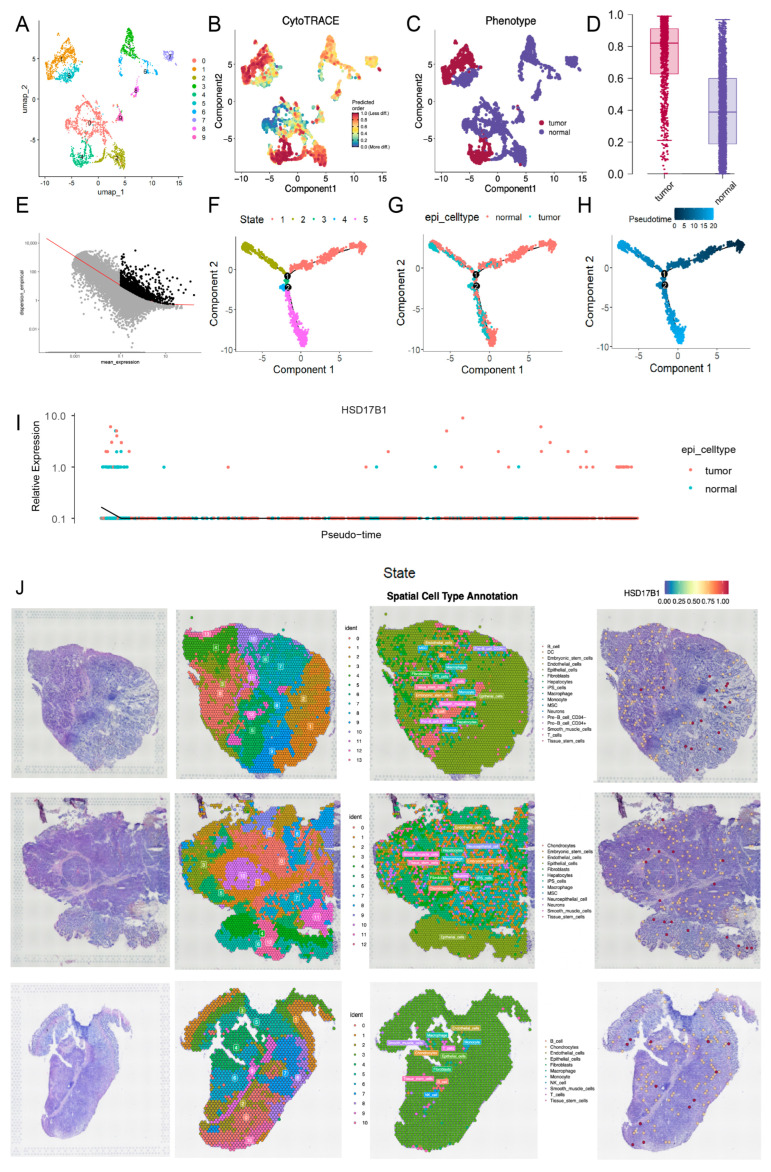
Pseudotime trajectory analysis and spatial transcriptome (ST) analysis. (**A**) UMAP clustering plot of 10 distinct subclusters of epithelial cells. (**B**) CytoTRACE score of epithelial cells. (**C**) Distribution of tumor-derived epithelial cells and normal epithelial cells divided according to the established CytoTRACE score. (**D**) Box plot of CytoTRACE score of tumor-derived epithelial cells and normal epithelial cells. (**E**) Trajectory construction of highly variable genes. (**F**) Cell state distribution of epithelial cells in pseudotime trajectory analysis. (**G**) Distribution of tumor-derived epithelial cells and normal epithelial cells in pseudotime trajectory analysis. (**H**) Time trajectory of epithelial cells in pseudotime trajectory analysis. (**I**) The expression of *HSD17B1* in pseudotime trajectory analysis. (**J**) Cluster annotation of spatial transcriptome (ST) analysis and the expression of *HSD17B1* in GSM7990475 (top), GSM7990477 (middle), GSM7990480 (bottom) samples of GSE251950.

To further explore cellular heterogeneity, epithelial cells were re-clustered into 10 distinct subclusters (Figure 9A). Integration with CytoTRACE scoring revealed that subclusters 1 and 4 were predominantly composed of tumor-derived epithelial cells, while the other subclusters represented normal epithelial populations (Figure 9B–D). Pseudotime trajectory analysis of epithelial cells revealed a stepwise differentiation process comprising five distinct stages, with an initial starting point, two divergent branches, and a transitional phase during mid-differentiation where normal cells begin to undergo malignant transformation (Figure 9E–H). Notably, *HSD17B1* expression was upregulated at the final stage of differentiation, indicating its progressive elevation during the malignant transformation of normal cells into tumor cells (Figure 9I; expression of other candidate genes is shown in Figure S6). ST analysis of three samples (GSM7990475, GSM7990477, and GSM7990480) partitioned the samples into 14, 13, and 11 distinct clusters, respectively, and annotated them into 17, 13, and 11 cell types. Commonly identified cell types across the samples included epithelial cells, endothelial cells, macrophages, and fibroblasts (Figure 9J). Expression analysis showed that *HSD17B1* was predominantly localized in epithelial cells, consistent with the scRNA-seq results, and its generally elevated expression suggested a potential association with tumor progression.

### 2.7. Heterogeneity of the Tumor Microenvironment (TME) by ST Analysis

ST sequencing results revealed the distribution characteristics of spatial transcript counts (nCount_Spatial) (Figure S7A). The spatial heatmap showed that there were significant regional differences in transcript counts within the tissues, and regions with high transcript counts were mainly concentrated in tumor cell-enriched areas. Meanwhile, the violin plot further verified the differences in overall transcript counts among different tissue samples and the grouped violin plot revealed that uneven distribution of transcript counts within each spatial cluster, indicating high heterogeneity within gastric cancer tissues. In the distribution of each spatial cluster in tissue sections, different spatial clusters exhibited distinct spatial localization characteristics in tissue sections: some clusters were mainly distributed in the tumor margin area, while others were localized in the tumor core area or stromal area (Figure S7B–D). This spatial distribution pattern indicated that tumor tissues are composed of multiple cell populations, and these cell populations maintain relatively independent histological characteristics in spatial structure.

Uniform manifold approximation and projection (UMAP) dimensionality reduction results demonstrated that each GC tissue sample could be divided into multiple cell clusters (Figure S8A). After cell type annotation, it was found that these cell clusters mainly included epithelial cells, fibroblasts, immune cells (e.g., B cells, T cells, and macrophages), with a small number of endothelial cells. Further cluster annotation results revealed that different cell types had a certain consistency among samples, but also showed inter-individual differences. Additionally, pseudotime analysis presented the dynamic distribution characteristics of different cell clusters in the trajectory graph (Figure S8B–D). The trajectory structure showed that epithelial cells were located at the starting position of the main differentiation path during tumor progression, while fibroblasts and immune cells aggregated in the later stage of the trajectory, suggesting that these two types of cells may play key roles in the formation of the TME and immune regulation. In addition, the three samples showed significant differences in trajectory structure and cell distribution pattern, further confirming the high heterogeneity of TME.

## 3. Discussion

Recent molecular studies have demonstrated that genistein, a soy isoflavone, affects signaling pathways associated with GA. It modulates inflammatory markers like NF-κB and cell cycle regulators such as Cyclin D and p53, while its tumor-suppressive effects are mediated through molecular markers like PCDH17 and SOX2 [13,23]. Cell-based studies have identified genistein-interacting molecules with potential anti-cancer properties [24], and bioinformatics approaches have been employed to further explore genistein’s molecular mechanisms in combating GA. In our integrative analysis, six candidate key genes (*HSD17B1*, *CCNB1*, *CCNB2*, *EZH2*, *CDKN2A*, and *IGFBP6*) were identified as potential targets for genistein’s therapeutic effect in GA.

Both *CCNB1* and *CCNB2*, members of the cyclin B family, encode Cyclin B1 and Cyclin B2, which interact with CDK1 to regulate the G2/M transition, acting as essential drivers of mitotic entry [25,26]. Activation of upstream pathways such as PI3K/Akt enhances cell cycle progression by increasing Cyclin B expression or activity [27]. Overexpression of these cyclins has been consistently associated with heightened tumor proliferation, increased malignancy, and poor prognosis in GC [28]. Genistein inhibits mitosis by inducing cell cycle arrest and downregulating *CCNB1* expression, further supporting its role as a therapeutic target [14,29].

*EZH2*, the catalytic subunit of Polycomb Repressive Complex 2 (PRC2), is a histone methyltransferase that catalyzes H3K27 trimethylation and transcriptional repression. As an oncogenic driver in various cancers, *EZH2* promotes tumorigenesis and sustains cancer stem cell maintenance [30]. It has been recognized as a novel therapeutic target in GA [31]. Notably, cancer-derived exosomes facilitate glycolysis, proliferation, and metastasis through the miR-198/EZH2 axis [32]. In oral squamous cell carcinoma, genistein selectively induces tumor cell apoptosis by suppressing the PI3K/AKT–EZH2 signaling pathway, underscoring its potential as a targeted therapeutic agent [33].

*CDKN2A* (cyclin-dependent kinase inhibitor 2A) is a tumor suppressor gene that induces cell cycle arrest at the G1/S and G2/M checkpoints [34,35]. However, this function appears inconsistent with our findings, which may be attributed to mutations that result in functionally defective *CDKN2A* [36]. Deng C et al. demonstrated that *CDKN2A* mutations (e.g., deletions, point mutations) in GA suppress interferon-α/γ responses, inflammatory reactions, and other immune-related pathways, impairing anti-tumor immunity and correlating with shorter patient survival [36]. This suggests that the paradoxical association between high *CDKN2A* expression and poor prognosis in our study may result from the predominance of mutant *CDKN2A* in highly expressed cases (e.g., loss of p16-mediated cell cycle inhibitory function). Here, high expression merely represents a concomitant phenomenon of mutation, whereas the mutation itself is the key driver of disease progression and impairment of its tumor-suppressive function. Additionally, a study in renal carcinoma demonstrated that genistein suppresses tumor cell proliferation by enhancing *CDKN2A* expression through promoter hypomethylation [37], suggesting an epigenetic mechanism that may underlie CDKN2A activation in certain cancers.

*IGFBP6*, a member of the insulin-like growth factor binding protein family, activates the MAPK signaling pathway and enhances cell migration [38]. *IGFBP6*, a specific inhibitor of insulin-like growth factor II (IGF-II), exhibits significantly stronger binding capacity to IGF-II than to IGF-I and can effectively inhibit the proliferation of malignant tumors with high IGF-II expression [39]. Meanwhile, similar to other members of the IGFBP family, *IGFBP6* can also regulate the activity of cancer cells directly without relying on the IGF signaling pathway. In vitro studies [39,40] have confirmed that this protein can block the angiogenesis response mediated by vascular endothelial growth factor. In conclusion, *IGFBP6* exhibits dual functional characteristics in cancer regulation, and its specific mechanism of action remains to be further explored. While no previous evidence supports a direct interaction between *IGFBP6* and genistein, our findings provide novel insights by identifying *IGFBP6* as a potential target of genistein.

*HSD17B1* (17beta-hydroxysteroid dehydrogenase type 1), identified as a key gene in this study, is central to our discussion. Belonging to the short-chain dehydrogenase/reductase (SDR) family, *HSD17B1* plays a pivotal role in steroid metabolism [41]. It significantly enhances androgenic effects, although its catalytic efficiency for converting androstenedione to testosterone is lower than its conversion of estrone (E1) to estradiol (E2) in vitro [42,43]. Notably, transgenic overexpression of human *HSD17B1* in mice results in a masculinized phenotype in females, highlighting its substantial impact on androgen-related pathways in vivo [44]. Given the higher incidence of GC in males than females, it is plausible that androgen-related risk factors may contribute to this sex disparity in disease prevalence [45]. Additionally, high *HSD17B1* expression has been reported to correlate with poor prognosis in patients with GC [46], which aligns with our findings. At the single-cell level, *HSD17B1* was predominantly expressed in malignant epithelial clusters, and ST revealed its localization in tumor-enriched regions, providing robust spatial and cellular context to its pathogenic role. Collectively, these multi-omics results validate *HSD17B1* as a genistein-responsive gene and reinforce its biological relevance in GC progression, supporting its potential as a therapeutic target.

GSEA revealed that multiple candidate key genes were significantly enriched in pathways related to the cell cycle, olfactory transduction, and MAPK signaling. Dysregulation of the cell cycle is a hallmark of cancer initiation and progression [47]. Cyclins and cyclin-dependent kinases (CDKs), key regulators of cell cycle transitions, have emerged as promising therapeutic targets across various malignancies [48]. In GA, several cell cycle-related genes have demonstrated prognostic significance. Specifically, overexpression of *ESPL1* and *MCM5* has been significantly correlated with advanced tumor stages and disease progression, underscoring their potential utility as biomarkers for predicting clinical outcomes [49]. Though traditionally linked to sensory perception, olfactory transduction is increasingly implicated in tumor biology. This is through ectopic expression of olfactory receptors (ORs), GPCR-family proteins that activate intracellular cascades (e.g., cAMP, calcium flux, cytoskeletal remodeling), promoting cancer cell migration and invasion [50]. Notably, the functional role of ORs in tumors is not limited to pro-tumorigenic effects: activation of ORs within tumor cells holds the potential to substantially reduce tumor proliferation or even bring tumor cell growth to a complete halt [50]. Previous research has suggested that GC may be associated with the ectopic expression of ORs, which are aberrantly activated in non-olfactory tissues and participate in tumor-related signaling pathways [51]. Mechanistically, key molecules governing olfactory signal transduction have been identified as potential modulators of this OR-mediated tumor regulatory pathway. Specifically, genistein exerts its effects by altering the phosphorylation status of target proteins—a process indispensable for the proper functioning of olfactory signal transduction, which may in turn modulate OR-dependent regulatory effects on tumor biology. The MAPK signaling pathway, recurrently enriched in several genes, regulates diverse cellular processes such as growth, differentiation, and apoptosis, and is frequently dysregulated in GC [52]. These findings suggest that the candidate genes may contribute to tumor progression through the cell cycle, olfactory transduction, and MAPK signaling pathways, thereby elucidating the mechanism of genistein in treating GA.

The results from scRNA-seq and ST analyses identified epithelial cells as a key cell type in GA. Pseudotime analysis revealed a progressive upregulation of *HSD17B1* expression specifically during the transition of epithelial cells from normal phenotypes to malignant tumor cells. Epithelial cells perform a variety of vital physiological functions in the human body, acting as a physical barrier to protect underlying tissues from external insults while also participating actively in processes such as absorption and secretion. By forming tight junctions, epithelial cells regulate selective permeability across tissue interfaces, thereby maintaining internal homeostasis [53]. Additionally, epithelial cells contribute to innate immune responses by detecting and responding to microbial invasions [54]. In GA, these critical functions are disrupted. The malignancy is driven by transformed epithelial cells that exhibit uncontrolled proliferation and metastatic potential, largely facilitated by EMT [55,56]. Key molecular drivers of EMT include non-coding RNAs such as the long non-coding RNA CHRF and microRNA miR-665 [57,58]. The stepwise upregulation of *HSD17B1* during epithelial transformation suggests its potential involvement in GA pathogenesis, possibly through disruption of epithelial integrity or modulation of EMT pathways. Epithelial cells have been demonstrated to detect signals derived from microorganisms, allergens, and tissue surface damage, and subsequently relay these signals to immune cells [59]. Given the pivotal role of epithelial cells in GA, we further utilized ST analysis to investigate the heterogeneity within the GA immune microenvironment. Our findings indicate that GA tumor tissues consist of diverse cell populations and exhibit distinct histological features with well-defined spatial organization. Pseudotime analysis further underscores the high degree of heterogeneity present in the TME of GA. These findings further highlight the importance of in-depth exploration of the TME in clarifying the development mechanism of GA.

This study synergistically integrated network pharmacology and bioinformatics approaches to systematically characterize the therapeutic targets and multifaceted anti-tumor mechanisms of genistein in GA. scRNA-seq revealed unprecedented cellular heterogeneity and dynamic state transitions within tumor ecosystems, while ST mapping accurately localized molecular expression patterns within their native tissue architecture. The network pharmacology framework further constructed a comprehensive “drug-target-pathway-disease” interaction network, collectively enabling a systematic dissection of tumor pathogenesis and treatment at both single-cell resolution and spatially resolved dimensions.

Notably, this study primarily focuses on descriptive correlation analyses and has limitations in several critical areas: the cohort sizes of the scRNA-seq and ST datasets are relatively limited. This limitation may lead to insufficient representativeness of the research results and introduce potential sampling bias, thereby affecting the generalizability of the conclusions to a certain extent. Therefore, in future research, we plan to further expand the size of the sample cohort and enhance the reliability and extrapolation value of the research results through validation in a larger scope. The mechanistic interpretation of the observed molecular changes is preliminary; causal relationships have not been experimentally validated through targeted functional studies, and clinical translatability requires more robust evidence from patient-derived models. Additionally, incomplete characterization of tumor microenvironmental crosstalk and potential sampling biases may affect the generalizability of the data. Current research has not verified *HSD17B1* as a genistein target via in vitro or in vivo functional experiments, and this missing verification leaves the drug-target causal relationship without direct experimental support. Although the association between *HSD17B1* and malignant epithelial transformation has been fully supported by scRNA-seq and ST analyses, its relevance to therapeutic translation still requires verification through functional experiments. To address these gaps, future investigations should prioritize mechanistic validation, preclinical testing, and multicenter clinical studies to evaluate therapeutic efficacy across diverse populations. These efforts will significantly enhance the translational potential of our findings for precision oncology applications.

## 4. Materials and Methods

### 4.1. Data Collection

The scRNA-seq dataset GSE264203 (platform: GPL24676) contains malignant epithelial cells from 5 patients with GA [60], while 3 GA samples (GSM7990475, GSM7990477, and GSM7990480) from GSE251950 (platform: GPL24676) were selected for ST analysis [61]. The bulk RNA sequencing datasets GSE29998 (platform: GPL6947) [62] consists of 50 tumor samples and 49 controls, and GSE118916 (platform: GPL15207) [63] includes 15 pairs of gastric tumor and adjacent non-tumor (normal) tissues. All datasets were sourced from the Gene Expression Omnibus (GEO) database (http://www.ncbi.nlm.nih.gov/geo/ (accessed on 11 October 2025)).

### 4.2. Acquisition of Drug Target Genes

Human protein targets for genistein were identified by querying the ChEMBL (https://www.ebi.ac.uk/chembl/ (accessed on 11 October 2025)), STITCH (http://stitch.embl.de/ (accessed on 11 October 2025)), and SwissTargetPrediction (http://www.swisstargetprediction.ch/ (accessed on 11 October 2025)) databases using its PubChem-derived chemical structure and SMILES notation (https://pubchem.ncbi.nlm.nih.gov/ (accessed on 11 October 2025)). The resulting target lists were merged and duplicates removed to identify drug target genes.

### 4.3. Identification of GA Target Genes

Disease-associated targets for GA were retrieved from established databases, including Online Mendelian Inheritance in Man (OMIM, https://omim.org/ (accessed on 11 October 2025)) and Genecards (https://www.genecards.org/ (accessed on 11 October 2025)), with a relevance score > 7. Duplicate entries were then removed to identify GA-related target genes. Principal component analysis (PCA) was performed on the GSE29998 dataset to assess the separation between sample groups. DEGs between GA and control samples in the GSE29998 dataset were identified using the limma (v3.64.1) package (|log2FC| > 1 and adjust. *p* < 0.05) [64].

### 4.4. Acquisition and Functional Analysis of Candidate Genes

To identify potential candidate genes related to GA treatment by genistein, the intersection of drug target genes, GA target genes, and DEGs was analyzed. A box plot was generated to visualize the expression patterns of these candidate genes across different experimental groups in the GSE29998 dataset. Chromosomal localization of candidate genes was determined using the circlize (v0.4.16) package [65]. Pearson correlation coefficients between these genes were calculated to assess their interrelationships. A PPI network was constructed using the GeneMANIA database (http://genemania.org/ (accessed on 11 October 2025)) to explore potential protein-level interactions among the candidate genes. To further elucidate the molecular functions and mechanisms of these genes, extensive functional enrichment analyses were conducted using the clusterProfiler (v4.16.0) package, incorporating GO and KEGG pathway annotations [66] (*p* < 0.05). The aPEAR (v1.0) package was employed to cluster the annotation results, facilitating the identification of underlying biological themes [67].

### 4.5. Machine Learning

To facilitate machine learning, the GSE29998 dataset was randomly divided into two subsets using a 7:3 split ratio, with the larger portion allocated for model training and the smaller portion reserved for validation. A range of machine learning algorithms, including Random Forest (RF), Extreme Gradient Boosting (XGBoost), Generalized Linear Model (GLM), Neural Network (NNET), Support Vector Machine (SVM), k-Nearest Neighbors (k-NN), Gradient Boosting Machine (GBM), NB, Adaptive Boosting (ADA), Least Absolute Shrinkage and Selection Operator (LASSO), Bootstrap Aggregating (BAG), and Decision Tree (DT), was implemented based on candidate genes using the caret (v7.0.1) package [68]. All models adopted 5-fold repeated cross-validation (train Control (method = “repeatedcv”, number = 5)) to evaluate the robustness and generalization ability of the algorithms. The model training parameters were set to default values, and the binary classification threshold used the default 0.5 cutoff for probability prediction. The positive class was defined as the control group, which served as the reference for all performance evaluations and class discrimination. For model performance evaluation, a unified framework for residual assessment and variable importance interpretation was constructed using the DALEX package (v2.0) [69]. Meanwhile, the performance of each model was evaluated through ROC curves, residual box plots, reverse cumulative distribution of residual graphs, and confusion matrices. The model with the highest AUC value, minimal residuals, and highest accuracy was selected as the optimal model, with the genes identified by this model considered as candidate key genes. Additionally, ROC curves for each candidate key gene were constructed in the GSE29998 dataset to assess their diagnostic efficacy. The GSE118916 dataset served as an external validation cohort to construct ROC curves for the prediction model based on candidate key genes, further assessing their diagnostic performance. Subsequently, samples from all datasets on the website (http://kmplot.com/analysis/index.php?p=service&cancer=gastric (accessed on 11 October 2025)), except the GSE62254 dataset, were stratified into high- and low-expression groups based on the optimal cutoff expression levels for each candidate key gene, and survival differences between these groups were analyzed on the website [70].

### 4.6. GSEA Enrichment Analysis

GSEA was performed to explore the potential biological pathways associated with each candidate key gene. GSEA was conducted using the “clusterProfiler” R package (v4.16.0) [66] and the org.Hs.eg.db gene set. Enrichment scores were computed with 1000 permutations, and pathways with nominal *p* < 0.05 and |NES| > 1 were considered significantly enriched. The top five enriched KEGG pathways for each gene were visualized to highlight their biological relevance.

### 4.7. Molecular Docking

Molecular docking simulations between genistein and candidate key gene-encoded proteins were conducted using the online tool CB-dock2 (https://cadd.labshare.cn/cb-dock2/php/blinddock.php (accessed on 11 October 2025)) [71]. Tertiary protein structures were retrieved from the PDB database (https://www.rcsb.org (accessed on 11 October 2025)) or AlphaFold2 (https://alphafold.ebi.ac.uk (accessed on 11 October 2025)) in PDB format, while genistein’s structure was obtained in SDF format from PubChem (https://pubchem.ncbi.nlm.nih.gov/ (accessed on 11 October 2025)). Proteins were preprocessed by adding non-polar hydrogens, assigning Gasteiger charges, and converting them into PDBQT format. Ligand structures were converted from SDF to PDB, followed by transformation into PDBQT files with defined rotatable bonds. Binding affinities were evaluated based on the calculated binding free energy (ΔG ≤ −5.0 kcal/mol). Based on these findings, the gene exhibiting the lowest binding energy to genistein was identified as the key gene.

### 4.8. MD Simulation

MD simulations were performed in GROMACS 2020 using the docked complexes of the key gene and genistein [72]. The protein was constructed using the CHARMM36 force field, and ligand parameters were generated with the Sobtop tool based on the GAFF force field. Atomic types, bond angle parameters, and charge distributions were checked to confirm compatibility with GROMACS. In the simulation, the TIP3P water model was used, and Na^+^/Cl^−^ ions were added to neutralize charges and simulate physiological conditions. Simulations were carried out in an NPT ensemble with a 2-fs time step, applying LINCS hydrogen bond constraints, Particle Mesh Ewald (PME) electrostatics (1.2 nm cutoff), a 1.0 nm van der Waals cutoff, and neighbor list updates every 10 steps. Temperature (300 K) and pressure (1 bar) were controlled using the V-rescale thermostat and Berendsen barostat. The system underwent energy minimization using the steepest descent, with convergence achieved when the maximum atomic force fell below 1000 kJ/mol/nm. This was followed by stepwise equilibration: 100 ps in the NVT ensemble and 100 ps in the NPT ensemble at 300 K. During both the NVT and NPT equilibration stages, convergence was determined by monitoring the system’s energy, temperature, and pressure. The equilibration was considered complete and transitioned to the next stage only when the curves of these parameters stabilized, showing no systematic drift and remaining within a narrow random fluctuation range. Production simulations were performed for 100 ns (NPT), with trajectory snapshots saved every 10 ps. Trajectory analysis was conducted using VMD 1.9.4 and PyMOL 3.1, and structural evolution was assessed through root mean square deviation (RMSD), radius of gyration (Rg), root mean square fluctuation (RMSF), buried solvent accessible surface area (SASA), and hydrogen bond dynamics.

### 4.9. scRNA-Seq Data Processing

Data filtering was conducted using the Seurat (v5.3.0) package on the GSE264203 dataset [73]. Quality control filtering excluded cells with the following characteristics: detected genes < 200 or >7500, UMI counts < 600, mitochondrial gene percentage > 10%, or hemoglobin gene percentage > 0.1%. Following logarithmic normalization, the vst function was used to identify the top 2000 HVGs showing maximal inter-cellular variation. PCA was performed on these HVGs, with significant PCs selected through integrated assessment of elbow plot inflection points and JackStraw permutation *p*-values (*p* < 0.05). Using these components, cell clustering was generated via the FindNeighbors and FindClusters functions at resolution = 0.5, followed by t-SNE dimensionality reduction. Cluster identities were annotated by cross-referencing differentially expressed marker genes with established literature signatures [60]. The expression patterns of key genes across different cell types were systematically analyzed. Additionally, a systematic analysis of the subcellular localization of key genes was performed using the Genecards database. Tumor cells were distinguished from non-neoplastic cells using InferCNV (v1.20.0) [74], with immune cells from the same sample serving as the reference group. Cells designated as “Malignant” by the HMM model and exhibiting large-scale chromosomal aberrations were classified as malignant. The quantification of cellular HSD17B1 expression levels in each cell type was conducted through AUCell scoring, followed by stratification of cells into high- and low-expression subgroups using median AUCell scores as the threshold. Pathway enrichment analysis was then performed using single-sample GSEA (ssGSEA), systematically interrogating the Hallmark gene sets curated in MSigDB (https://www.gsea-msigdb.org/gsea/msigdb (accessed on 11 October 2025)). Correlations between cell types and pathways were determined, followed by visualization of pathways showing significant enrichment.

### 4.10. Secondary Clustering and Pseudotime Trajectory Analysis

Cell types exhibiting distinct expression patterns of key genes were selected for secondary clustering analysis. The CytoTRACE algorithm was then employed to compute stemness scores for cells, which were subsequently used to classify cells into tumor and normal categories.

Additionally, pseudotime trajectory analysis was conducted using the monocle (v2.36.0) package to further investigate the functional dynamics and developmental processes of these cell types or states during disease progression, as well as the expression patterns of key genes [75].

### 4.11. ST Analysis

ST analysis in GSE251950 was performed using the Seurat package (v4.3.0). For each sample, the gene-spot matrix was normalized with SCTransform to adjust for technical variability and stabilize feature variances. HVGs were identified using the FindVariableFeatures function, followed by data scaling with the ScaleData function. PCA was performed using the RunPCA function. Clustering was conducted via the Louvain community detection algorithm on a shared nearest-neighbor graph of the first 30 PCs constructed by the FindNeighbors and FindClusters functions (resolution parameter = 0.8). Cluster-specific marker genes were identified using the FindAllMarkers function (min.pct = 0.1, logfc.threshold = 1). Automated cell type annotation was carried out by integrating Seurat-derived cluster marker genes with reference-based predictions from SingleR (v2.10.0) [76]. The spatial distributions of clusters and key gene expression patterns were visualized using the SpatialFeaturePlot function.

### 4.12. TME Analysis in ST

10x Genomics ST data were imported using the Read10X_h5 function, while spatial images and positional information were loaded via the Read10X_Image function. Subsequently, a Seurat object was created, and spatial images were attached to this object to complete data preprocessing. Data normalization was performed using SCTransform, followed by dimensionality reduction via PCA. Neighbor search and cluster analysis were then conducted, and the results were visualized using UMAP.

DEGs were identified using the FindAllMarkers function and filtered based on log fold change (logFC) and adjusted *p*-values. Spatial feature plots and violin plots were generated to visualize the spatial distribution of distinct marker genes and their expression levels, respectively. Additionally, spatially variable gene analysis was performed to explore genes with expression patterns that vary across spatial locations. Automatic cell type annotation was implemented using the SingleR package combined with reference datasets from celldex. Each cell was assigned a corresponding cell type, and the annotation results were visualized on both UMAP plots and spatial distribution maps to confirm the spatial localization of different cell types.

Spatial trajectory construction was conducted using the monocle3 package, following the steps below: (1) The Seurat object was converted to a cell_data_set (CDS) object (the core data structure of monocle3) using as.cell_data_set, and Seurat-derived cluster information was added to the colData of the CDS object. (2) Cells were clustered using the cluster_cells function, and the cell trajectory graph was acquired via the learn_graph function. (3) Pseudotime values for each cell were calculated using the order_cells function. (4) The computed pseudotime information was added back to the original Seurat object, and spatial visualization plots were generated to display the spatial distribution of pseudotime.

### 4.13. Statistical Analysis

Statistical analyses were performed in R (v4.5.0) and Python (v3.8.10). Comparisons of continuous variables between groups were made using Wilcoxon tests, with statistical significance set at *p* < 0.05.

## 5. Conclusions

In conclusion, this study systematically identified therapeutic targets of genistein in GA through an integrated approach combining network pharmacology, transcriptomic analysis, molecular docking, and MD simulations. HSD17B1 was identified as a potential core target, with its specific expression pattern observed in epithelial cell subsets, highlighting its functional relevance in tumor progression. The integration of scRNA-seq and ST data provided a comprehensive cellular and spatial map of genistein’s potential action landscape in GA, contributing novel insights into the study of drug mechanisms in this context.

## Data Availability

The scRNA-seq dataset GSE264203, GSE251950 and the bulk RNA sequencing datasets GSE29998, GSE118916 were sourced from the Gene Expression Omnibus (GEO) database (http://www.ncbi.nlm.nih.gov/geo/ (accessed on 11 October 2025)).

## Supplementary Materials

The following supporting information can be downloaded at https://www.mdpi.com/article/10.3390/ijms262110369/s1.

## Abbreviations

GC	Gastric cancer
GA	Gastric adenocarcinoma
scRNA-seq	Single-cell RNA sequencing
ST	Spatial transcriptomics
RNA-seq	RNA sequencing
MD	Molecular dynamics
GEO	Gene Expression Omnibus
DEGs	Differentially expressed genes
PCA	Principal component analysis
GO	Gene Ontology
KEGG	Kyoto Encyclopedia of Genes and Genomes
PPI	Protein–protein interaction
ML	Machine learning
RF	Random Forest
XGBoost	Extreme Gradient Boosting
GLM	Generalized Linear Model
NNET	Neural Network
SVM	Support Vector Machine
k-NN	k-Nearest Neighbors
GBM	Gradient Boosting Machine
NB	Naive Bayes
ADA	Adaptive Boosting
LASSO	Least Absolute Shrinkage and Selection Operator
BAG	Bootstrap Aggregating
DT	Decision Tree
ROC	Receiver operating characteristic
AUC	Area under the curve
GSEA	Gene set enrichment analysis
NES	Normalized enrichment score
PDB	Protein Data Bank
ΔG	Binding free energy
RMSD	Root mean square deviation
Rg	Radius of gyration
RMSF	Root mean square fluctuation
SASA	Solvent accessible surface area
HVGs	Highly variable genes
PCs	Principal components
t-SNE	t-distributed stochastic neighbor embedding
EMT	Epithelial–mesenchymal transition
ssGSEA	Single-sample GSEA
ORs	Olfactory receptors
GPCR	G protein-coupled receptor
CDKs	Cyclin-dependent kinases
CNAs	Copy number alterations
HMM	Hidden Markov model
