# Supplementary figures and images for: The Anti-Cancer Potential of Genistein: Single-Cell RNA Sequencing Analysis and Spatial Transcriptome Reveal That Genistein Targets HSD17B1 to Inhibit the Progression of Gastric Adenocarcinoma
Source: Int J Mol Sci. 2025 Oct 24;26(21):10369. doi: 10.3390/ijms262110369 (PMC12609499; doi:10.3390/ijms262110369)

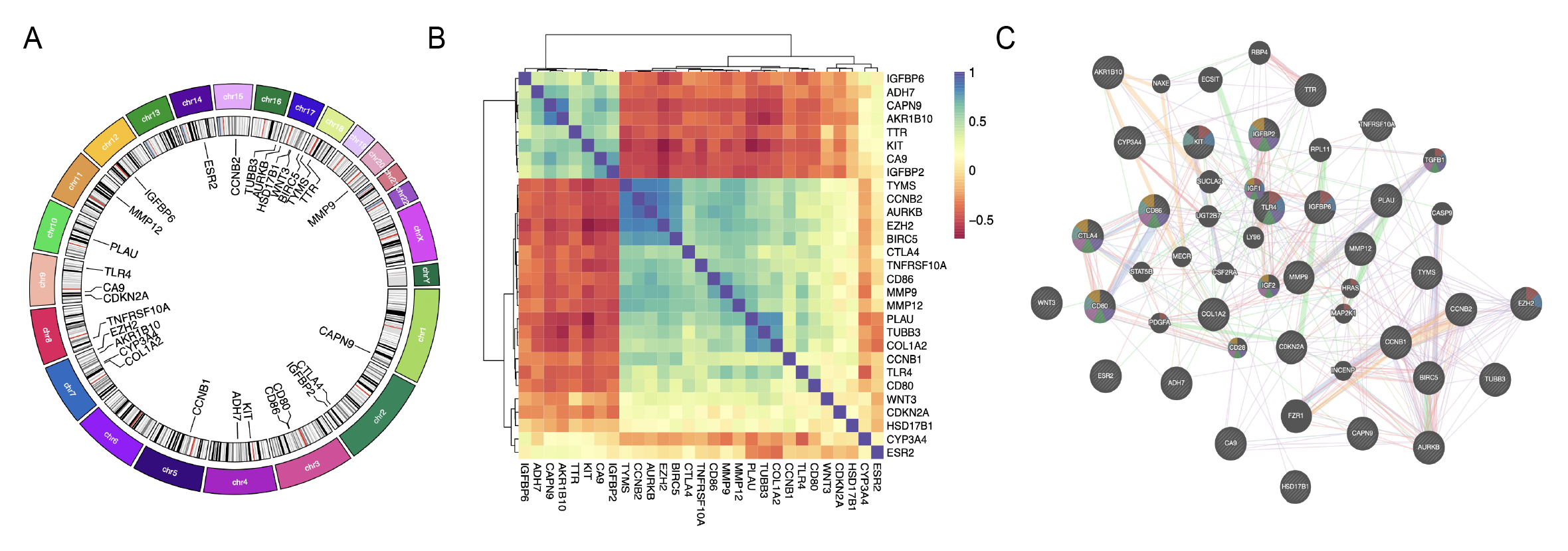

Supplement: Supplementary file 1 [file ijms-26-10369-s001.zip › ijms-3866917 Supplementary material/Figure S2.tif]

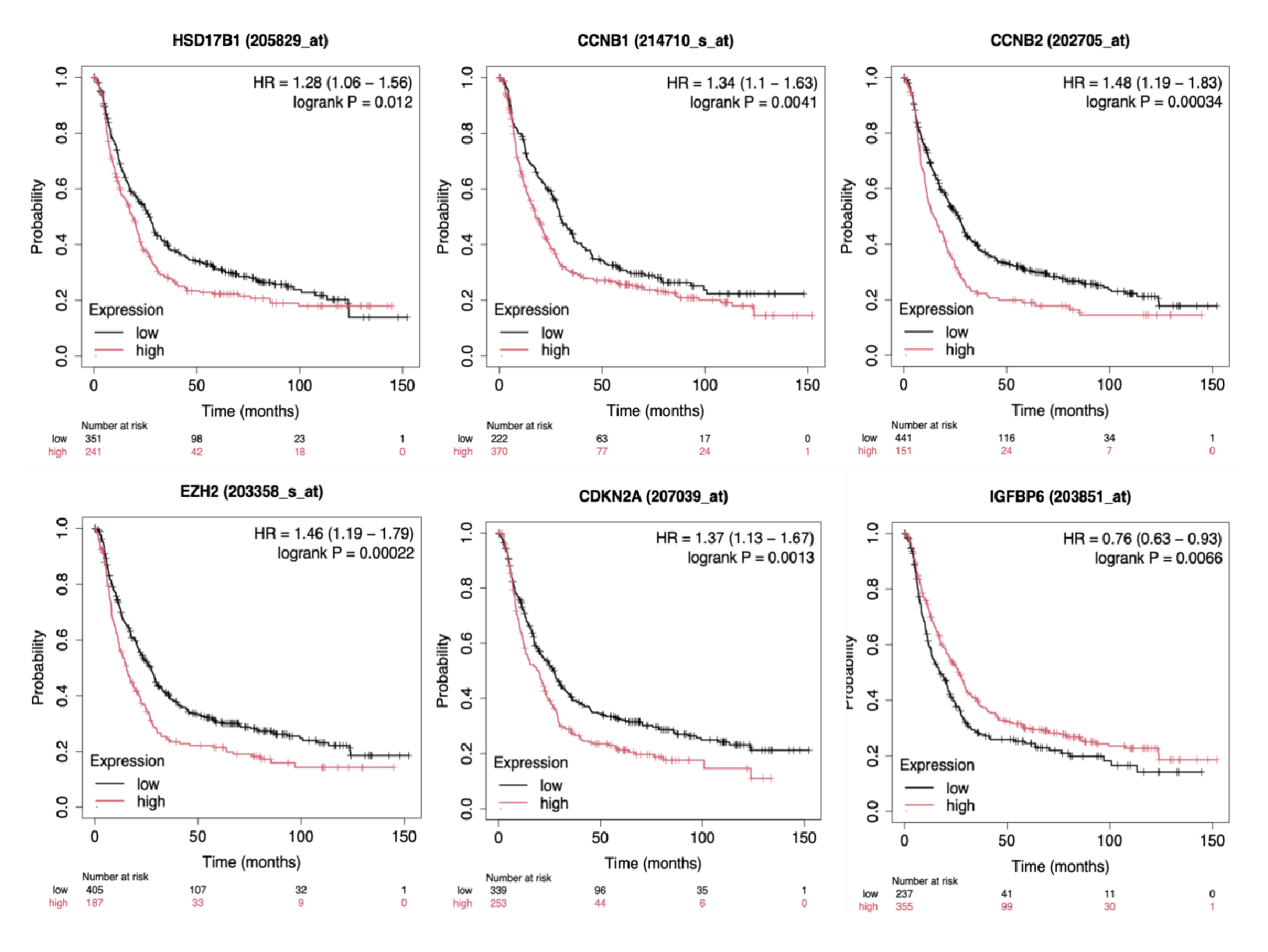

Supplement: Supplementary file 1 [file ijms-26-10369-s001.zip › ijms-3866917 Supplementary material/Figure S3.tif]

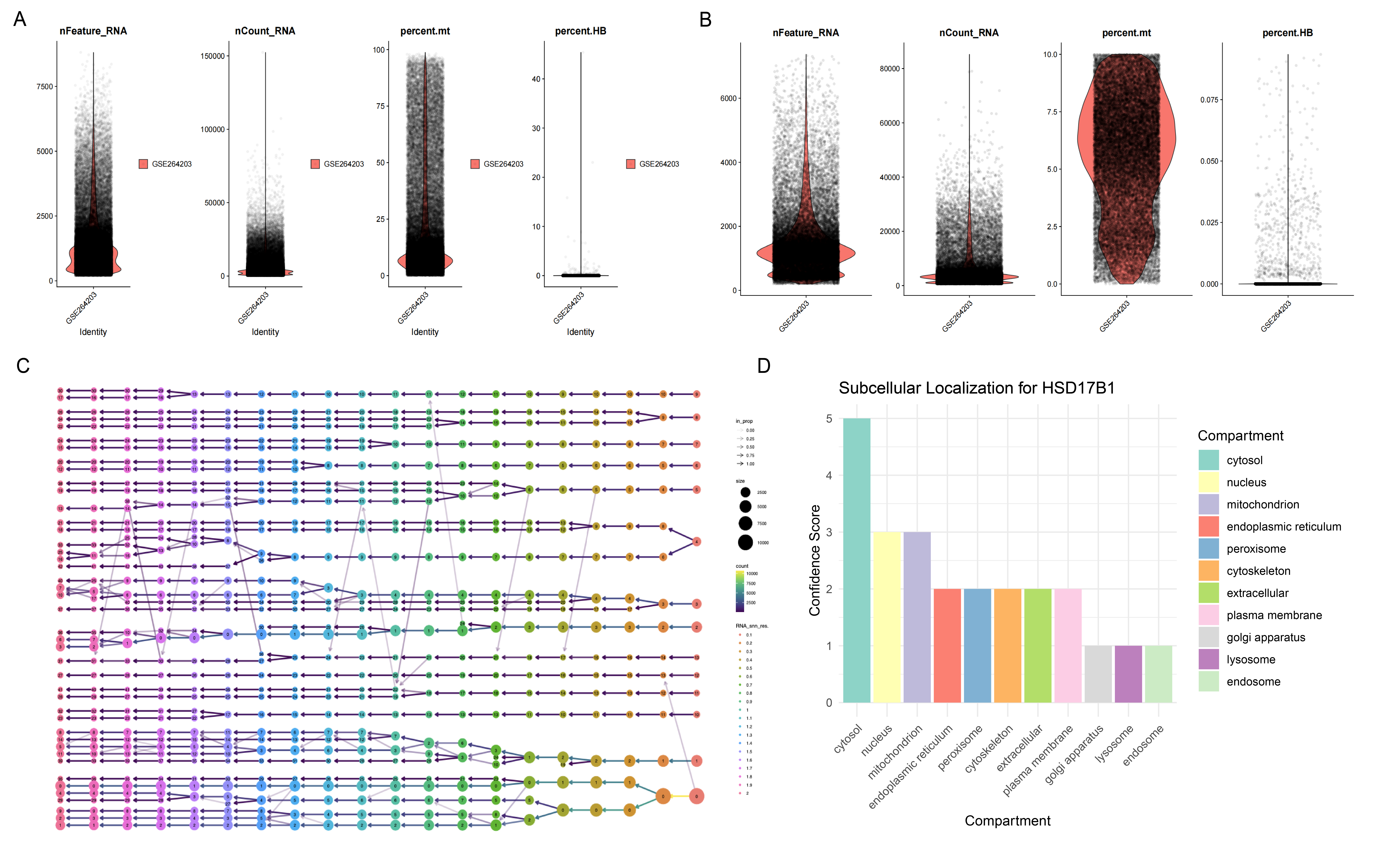

Supplement: Supplementary file 1 [file ijms-26-10369-s001.zip › ijms-3866917 Supplementary material/Figure S4.tif]

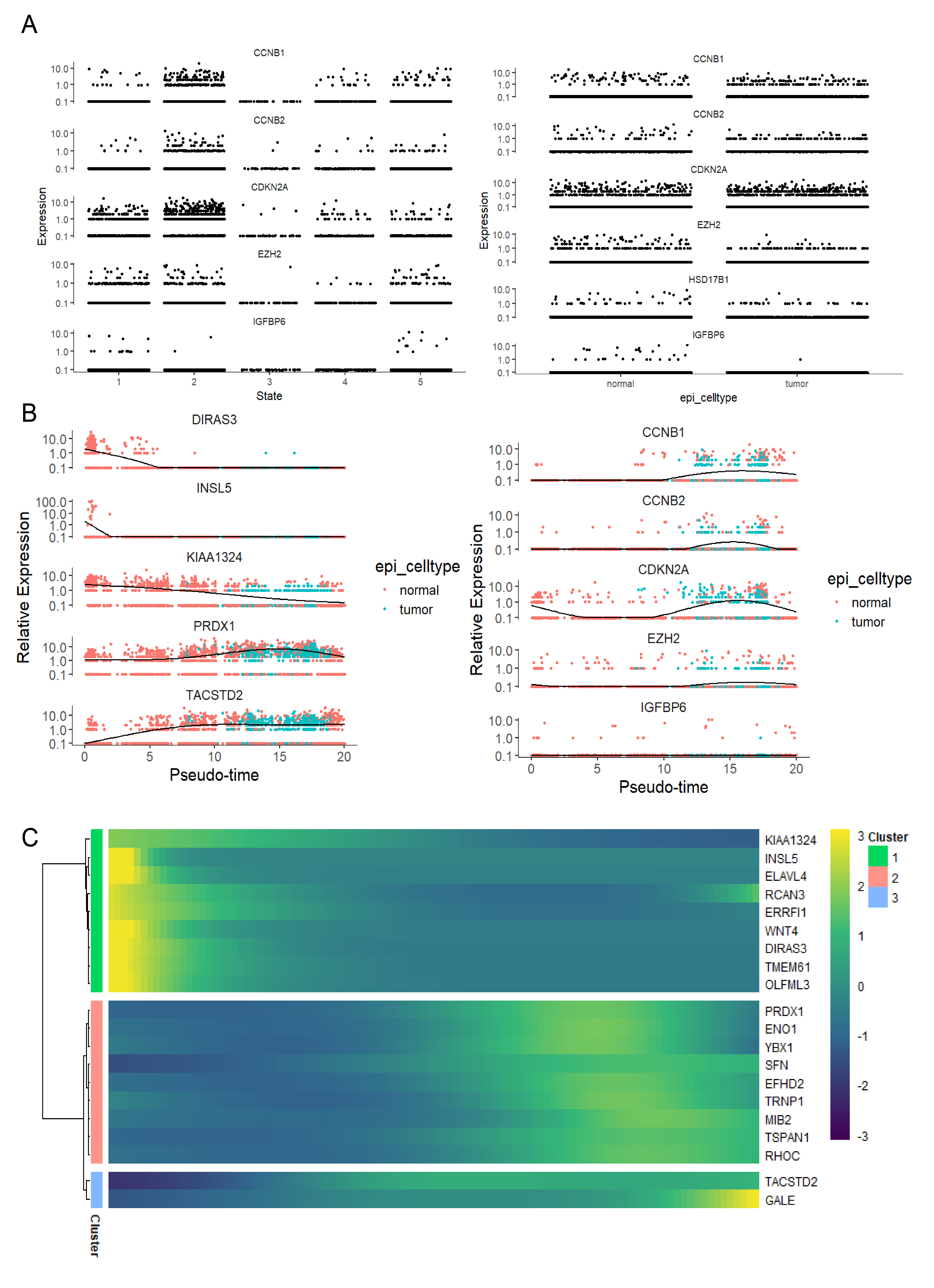

Supplement: Supplementary file 1 [file ijms-26-10369-s001.zip › ijms-3866917 Supplementary material/Figure S6.tif]
